# Application of Bayesian classification with singular value decomposition method in genome-wide association studies

**DOI:** 10.1186/1753-6561-3-s7-s9

**Published:** 2009-12-15

**Authors:** Soonil Kwon, Jinrui Cui, Shannon L Rhodes, Donald Tsiang, Jerome I Rotter, Xiuqing Guo

**Affiliations:** 1 Medical Genetics Institute, Cedars-Sinai Medical Center, 8635 West Third Street, Los Angeles, CA 90048, USA

## Abstract

To analyze multiple single-nucleotide polymorphisms simultaneously when the number of markers is much larger than the number of studied individuals, as is the situation we have in genome-wide association studies (GWAS), we developed the iterative Bayesian variable selection method and successfully applied it to the simulated rheumatoid arthritis data provided by the Genetic Analysis Workshop 15 (GAW15). One drawback for applying our iterative Bayesian variable selection method is the relatively long running time required for evaluation of GWAS data. To improve computing speed, we recently developed a Bayesian classification with singular value decomposition (BCSVD) method. We have applied the BCSVD method here to the rheumatoid arthritis data distributed by GAW16 Problem 1 and demonstrated that the BCSVD method works well for analyzing GWAS data.

## Background

Genome-wide association studies (GWAS) evaluate genetic variants throughout the entire genome with the goal of identifying susceptibility genes for diseases or conditions of interest. While a large number (*m*) of single-nucleotide polymorphisms (SNPs) are usually evaluated in a GWAS, sample size (*n*) is often limited due to substantial costs of recruitment and phenotype measurements. The fact that *m*>>*n *makes it unrealistic to analyze all SNPs simultaneously using traditional statistical methods, such as multiple linear regression analysis. It is therefore common for the analyses to be conducted one SNP at a time in GWAS. This means that 300,000 to 1,000,000 tests will be carried out for each GWAS study against each phenotype of interest. Such a large number of tests lead inevitably to a considerable problem with false positive results. To address this multiple testing issue in GWAS, two potential solutions have been investigated in recent years: evaluate false positive rates, e.g., Benjamini and Hochberg's false discovery rate and the *q*-value [1,2], or develop novel statistical methods for analyzing datasets where *m*>>*n *[3,4]. As one of the first proposed methods for analyzing multiple SNPs simultaneously when *m*>>*n*, we introduced the iterative Bayesian variable selection (IBVS) method [3]. Although sufficient to produce accurate and reliable results, the IBVS method has one barrier to efficient use: a relative long run time for GWAS data sets. To further improve the running speed, Kwon and Guo developed a Bayesian classification with singular value decomposition (BCSVD) method [4]. As a comparison, we applied the BCSVD method to the same sub-samples of the simulated rheumatoid arthritis (RA) data provided by Genetic Analysis Workshop (GAW) 15 Problem 3 and successfully identified the common genetic variants associated with RA status. Using exactly the same computer and the same data, we found that the run-time for BCSVD is less than half compared to that required for the IBVS method [4]. We applied the BCSVD method here to the GWAS data for RA sample provided in GAW16 Problem 1.

## Methods

### The BCSVD method

The BCSVD method used a binary probit model. Assuming that a latent variable is described by a linear regression model, the binary probit model can be expressed as:

where *z*_*n *× 1 _is a vector of latent variables, *X*_*n *× *p *_is the design matrix, *β*_*p *× 1 _is a vector of parameters to be estimated, and I_n _is an *n *× *n *identity matrix. By applying singular value decomposition (SVD) to the design matrix, X' = ADF', the model in (1) with the SVD of X can be written as

where L = FD and . Expressed as a linear combination of the original parameters (*β*), we call *γ *a superfactor vector. The joint distribution of *γ *and z can be expressed as the product of the prior distribution of *γ *and the likelihood function of z given *γ*, i.e., p(*γ*, z) ∝ p(*γ*)p(z|*γ*). The joint posterior distribution of *γ *and z given y can be written by multiplying p(*γ*, z) with the likelihood function of y given *γ *and z. By integrating out z and *γ*, respectively, from p(*γ*, z|y), we can have the posterior distributions of z and *γ*, respectively. With these posterior distributions, we can fit the model using Markov chain Monte Carlo with Gibbs sampler. The 95% credibility interval was used to check for the convergence of sampler. To transform *γ *back to *β*, we used the most general solution form for the linear equation (*γ *= *A'β*) and achieved the unique solution for *β *by choosing the generalized inverse of A' as A [5]. The test statistic for association was generated by permutation. Let  (*i *= 1, ⋯, *p*) be the estimate of i^th ^SNP effect from the raw data and  be the estimate of *i*^th ^SNP effect from the *j*^th ^shuffled. Let us define  as the difference between  and . Then, under the null hypothesis (*H*_0_: *β*_*i *_= 0), the statistic Λ_*i *_follows the standard normal distribution when k is large: , where  is the sample mean of  values, *j *= 1, ⋯, *k*, and se() is the standard error of . With the statistic Λ_*i *_(*i *= 1, ⋯, *p*), we provided the *p*-value to reject the null hypothesis.

### Association analysis

The evaluation of the BCSVD method for association analysis was performed in two steps. As the first step, we performed a genome-wide single SNP association analysis using the logistic regression model option in PLINK. The PLINK analysis results served for two purposes, one was for the comparison with the results from BCSVD method, and the other was for the selection of genomic regions. Even though the BCSVD method can be applied to the whole genome-wide association data, the requirement on computer memory is still a limiting factor. We therefore focused on chromosome regions selected through PLINK analysis results in our BCSVD analysis.

### Study sample

We used the whole-genome association data of the North American Rheumatoid Arthritis Consortium (NARAC) in GAW16 Problem 1. There were 2,062 subjects in the study, including 868 cases and 1,194 controls. Quality control on genotype data was performed with PLINK software. We eliminated 133,616 SNPs that failed the following quality control criteria: *p*-value < 10^-5 ^for Hardy-Weinberg equilibrium (HWE) test, minor allele frequency <1%, or missing data >10%. As a result, 411,464 SNPs were included in the PLINK association analysis.

### Imputation

For BCSVD analysis, we first selected chromosomes that have SNPs with *p*-value < 10^-7^. The best SNP on each chromosome that has the smallest *p*-value was identified. SNPs within 2 Mb upstream and downstream of the best SNP were then selected. Software MACH 1.0 [6] and HapMap phase 3 genotype data for the HapMap CEU sample were used for genotype imputation. Imputed SNPs with a squared correlation with true genotypes (*r*^2^) < 0.3 were excluded.

### BCSVD study sample

Analyzing all selected SNPs simultaneously for 2,062 samples requires tremendous computer memory that our current computers cannot yet handle. We therefore generated two data sets based on the imputed data: one had 1,000 subject (500 cases and 500 controls) randomly selected from 868 cases and 1,194 controls; the other had 200 subjects (100 cases and 100 controls) randomly selected from the above selected 1,000 subjects.

## Results

### Step 1. Single SNP association from GWAS

**Figure 1:**
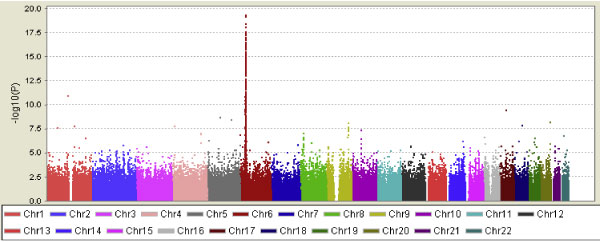
**GWAS analysis results of RA data from PLINK**. *x*-axis: Chromosomes 1-22; *y*-axis: -log10(*p*).

GWAS analysis results from PLINK were summarized in Figure 1. Nine chromosomal regions (1, 4, 5, 6, 9, 10, 17, 18, and 20) were identified based on our first step selection criteria of *p *< 10^-7^. The best peak was observed on chromosome 6, followed by chromosomes 1, 17, 5, 20, 9, 18, 4, and 10.

### Step 2. Evaluating multiple SNPs simultaneously with the BCSVD method

**Figure 2:**
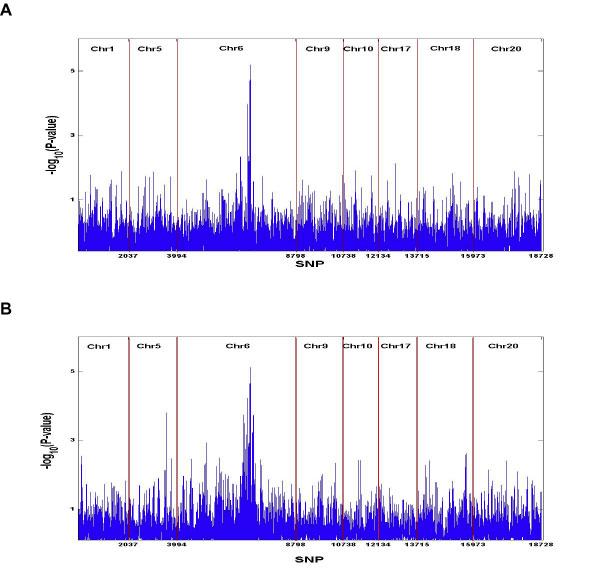
**Association analysis results from BCSVD method**. a, BCSVD association analysis results for 1,000 subjects. *x*-axis: SNPs in the 8 selected regions were numbered from 1 to 18,728. *y*-axis: -log_10_(*p*-value). b, BCSVD association analysis results for 200 subjects. *x*-axis: SNPs in the 8 selected regions were numbered from 1 to 18,728; *y*-axis: -log_10_(*p*-value).

Nine chromosomes (1, 4, 5, 6, 9, 10, 17, 18, 20) were identified that had SNPs with *p*-value < 10^-7^, we used 8 (all except chromosome 4) in the BCSVD analysis due to time limitation and extensive time required for imputation. A total of 18,728 SNPs, with 2037, 1957, 4804, 1940, 1396, 1581, 2258, and 2755 for chromosome 1, 5, 6, 9, 10, 17, 18 and 20, respectively, were evaluated simultaneously in BCSVD analysis for datasets with 200 and the 1,000 samples. The association results were summarized in Figure 2, a and b, where the *y*-axis represents -log_10_(*p*-value) and the *x*-axis shows SNP numbers. The strongest signal was observed again on chromosome 6 for datasets with 200 and 1,000 samples. The association signals identified in the dataset with 200 samples is very similar with those from the 1,000 subject sample, except for chromosome 1 and 5. Significant associations were identified for all the 8 regions in both datasets.

## Conclusion

The BCSVD method was applied to RA case-control data from Problem 1 of GAW16 for 8 selected regions. When we evaluated the association between RA affection status and all SNPs in selected regions simultaneously using BCSVD, significant associations were detected for all the 8 chromosomal regions, and the highest peak was observed on chromosome 6, which were consistent with the PLINK results. Even though the magnitude of significance [-log_10_(*p*-value)] appeared smaller than those from PLINK, we have to keep in mind that we used only datasets with 200 and 1,000 samples, respectively, in the BCSVD analysis, compared to the 2,062 samples in PLINK. More importantly, we have successfully avoided multiple testing issues because we performed only one test by evaluating all SNPs simultaneously. Similar results were observed in the datasets with 200 samples and 1,000 samples. We therefore conclude that the BCSVD method is a practical method for identifying genetic determinants in GWAS when sample size is much smaller than number of markers (*m*>>*n*). The BCSVD method has been implemented in our BAMGAS (Bayesian analysis methods for genetic association studies) program. While we are still working on a web-based user-friendly version, an executive version of the software is available from the authors.

## List of abbreviations used

BCSVD: Bayesian classification with singular value decomposition; GAW: Genetic Analysis Workshop; GWAS: Genome-wide association studies; HWE: Hardy-Weinberg equilibrium; IBVS: Iterative Bayesian variable selection; NARAC: North American Rheumatoid Arthritis Consortium; RA: Rheumatoid arthritis; SNP: Single-nucleotide polymorphism; SVD: singular value decomposition

## Competing interests

The authors declare that they have no competing interests.

## Authors' contributions

SK participated in the design and analysis and drafted the manuscript. JC participated in data cleaning and GWAS analysis. SR helped with data analysis and manuscript writing. DT helped to manage the data and analysis. JIR helped to draft the manuscript. XG participated in its design and coordination and helped to draft the manuscript. All authors read and approved the final manuscript.
